# Clinical profiling of TPOAb and TGAb in patients with thyrotrophin receptor antibody-negative thyroid eye disease: A single-center observational study in China

**DOI:** 10.3389/fendo.2025.1655598

**Published:** 2025-09-22

**Authors:** Gaojing Jing, Songbo Fu, Xinji Yang, Yueyue Li, Rui Ma, Wei Wu, Xulei Tang

**Affiliations:** ^1^ Department of Endocrinology, The First Hospital of Lanzhou University, Lanzhou, China; ^2^ The First Clinical Medical College, Lanzhou University, Lanzhou, China; ^3^ Gansu Clinical Medical Research Center for Endocrine Diseases, Lanzhou, China; ^4^ Senior Department of Ophthalmology, 3rd Medical Center of Chinese People's Liberation Army General Hospital, Beijing, China

**Keywords:** thyrotrophin receptor antibody-negative, thyroid eye disease, clinical characteristics, anti-thyroglobulin antibody, clinical management

## Abstract

**Background:**

Thyrotrophin receptor antibody (TRAb)-negative thyroid eye disease (TED) constitutes a clinically significant subset of TED, yet its features remain inadequately characterized. This study characterizes the clinical features of TRAb-negative TED patients.

**Methods:**

In this cross-sectional study, conducted at a Chinese tertiary hospital, 86 TRAb-negative TED patients underwent comprehensive ocular examinations and thyroid function tests. Clinical characteristics were systematically analyzed.

**Results:**

Of the 86 patients (mean age 45.24 ± 10.78 years; 53.5% female), 86.0% (n=74) exhibited bilateral ocular involvement. The primary manifestations included proptosis, eyelid edema, and eyelid retraction. The TPOAb (-)/TGAb (+) subgroup demonstrated significantly higher frequencies of eyelid erythema and lagophthalmos, along with severe NOSPECS grading, reduced visual acuity, elevated orbital pressure, and marked soft tissue involvement (all p<0.05). TPOAb-positive patients had significantly greater proptosis (p<0.05), while TGAb-positive patients showed an increased incidence of eyelid erythema (p<0.05). TGAb was identified as an independent risk factor for eyelid erythema (p<0.01).ROC analysis for TGAb predicting eyelid erythema yielded an AUC of 0.851 (sensitivity 0.778, specificity 0.831) at 3.980 IU/mL.

**Conclusion:**

TRAb-negative TED displays distinct clinical features. TPOAb and TGAb levels are associated with specific manifestations, highlighting their potential value in the assessment and management of TRAb-negative TED.

## 1 Introduction

Thyroid eye disease (TED) is the most common orbital autoimmune disorder in adults, affecting up to 40% of patients with Graves’ disease (GD) (1). Its clinical manifestations, including eyelid retraction, proptosis, diplopia, and optic neuropathy, significantly reduce quality of life (2). Thyroid receptor antibody (TRAb) levels are strongly associated with the TED incidence and pathogenesis of TED (3), correlating with clinical features such as clinical activity score (CAS), conjunctival injection, caruncle edema, lagophthalmos, and chemosis (4, 5).

However, TED can also present in TRAb-negative patients, irrespective of TPOAb or TGAb status. These cases often present with milder initial manifestations (6), which are frequently overlooked clinically, thereby delaying intervention. Symptoms may include subtle eyelid edema or diplopia. While progression to more severe complications like headache or vision loss is rare (7–9), prompt recognition of TRAb-negative TED is crucial. Despite documented associations with Hashimoto’s thyroiditis (HT) (8, 9), systematic studies analyzing TRAb-negative TED remain limited.

This study characterizes the clinical features in 86 TRAb-negative TED patients and explores potential underlying mechanisms. Our objectives are to enhance clinical recognition and inform diagnostic and therapeutic innovations.

## 2 Materials and methods

### 2.1 Participants

**Figure 1:**
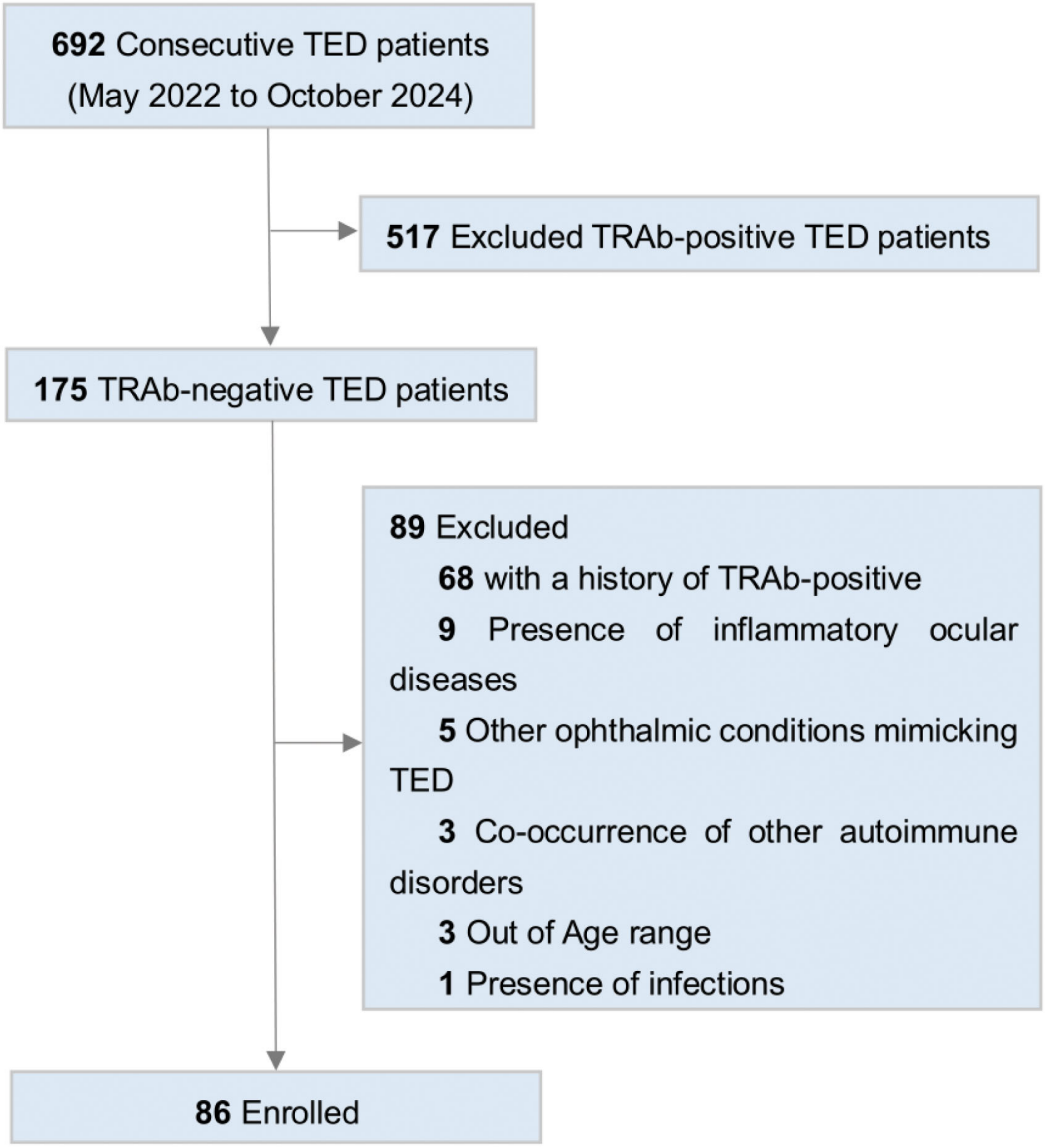
Flowchart depicting the patient screening process for enrollment.

This retrospective study included 86 TRAb-negative TED patients from the Senior Department of Ophthalmology, at the 3rd Medical Center of the Chinese PLA General Hospital. The inclusion criteria comprised: (1) TED diagnosis per Bartley’s criteria (10); (2) age 18–75 years; (3) confirmed TRAb-negative status. The exclusion criteria were: (1) Comorbid psychiatric, hematological, or non-HT autoimmune disorders; (2) Concurrent inflammatory ocular diseases, Immunoglobulin G4-related ophthalmic disease (IgG4-ROD), ocular trauma, infection, high myopia (> -6D), orbital tumors, severe myasthenia gravis, or TED-mimicking conditions; (3) History of TRAb positivity; (4) Pregnancy (**Figure 1**).

### 2.2 Ocular examinations

A single ophthalmologist administered a standardized protocol. To assess eyelid retraction, soft tissue involvement, proptosis [Hertel exophthalmometer; >16 mm threshold (11)], eyelid closure, diplopia [Gorman score (12)], visual acuity, intraocular pressure, orbital pressure, and optic nerve function. Disease activity was assessed using the Clinical Activity Score (CAS), with one point assigned foreach of the following signs: spontaneous orbital pain, gaze-evoked orbital pain, eyelid erythema, eyelid edema, conjunctival injection, chemosis, and caruncle edema. CAS ≥ 3 defined active disease; CAS < 3 defined inactive disease. Severity was graded according to EUGOGO guidelines (2) using the NOSPECS scoring system (13).

### 2.3 Thyroid function assay

Fasting venous blood samples were collected from all patients. TRAb levels were measured using third-generation electrochemiluminescence (Cobas e411, Roche Diagnostics; negative ≤1.75 IU/L). Additional thyroid related parameters were quantified using chemiluminescence assay (Beckman DXI800): free triiodothyronine (FT3, 3.28-6.47 pmol/L), free thyroxine (FT4, 7.64-16.03 pmol/L), thyroid-stimulating hormone (TSH, 0.49-4.91 mIU/L), TPOAb (≤9 IU/mL), and TGAb (≤4 IU/mL). Patients were classified as TPOAb-positive (>9 IU/mL) or TPOAb-negative (≤9 IU/mL), and TGAb-positive (>4 IU/mL) or TGAb-negative (≤4 IU/mL). Thyroid functional status was categorized as follows: euthyroid (all parameters within reference ranges), hyperthyroid (elevated FT3 and/or FT4 or suppressed TSH), or hypothyroid (decreased FT3 and/or FT4 or elevated TSH).

### 2.4 Statistical analysis

Demographic and clinical characteristics are summarized in **Tables 1**, **2**. Continuous variables are expressed as mean ± standard deviation (SD) or median. Categorical variables were compared using Chi-square or Fisher’s exact tests. For two-group continuous comparisons, independent samples Student’s t-test was applied when normality (Shapiro-Wilk test) and homogeneity of variances (Levene’s test) assumptions were met; otherwise, the Mann-Whiteney U test was used. For three or more groups, one-way ANOVA was used under normality and variance homogeneity; when these assumptions were not met, the Kruskal-Wallis H test was employed, with significant results (P < 0.05) followed by Dunn’s post-hoc test with Bonferroni correction for pairwise comparisons. Logistic regression analyzed eyelid erythema risk factors. Spearman correlation was used to evaluate the relationship between Gorman diplopia scores and TGAb levels. Receiver operating characteristic (ROC) curves were used to evaluate the predictive value of TGAb for eyelid erythema, considering AUC > 0.5 with p < 0.05 as being statistically significant. All analyses were conducted using IBM SPSS Statistics 26 (IBM Corp., Armonk, NY), with statistical significance defined as p<0.05.

**Table 1:** Demographic characteristics of 86 TED patients.

Parameter	TED patients (n = 86)
Age (years)	45.24 ± 10.78
Gender (f/m, n, %)	46 (53.5)/40 (46.5)
TED duration (months)	23.5 (10.0, 40.3)
Hyperthyroidism duration (months)	11.5 (0.0, 39.5)
Hyperthyroidism with a TRAb-negative history (n, %)	53 (61.6)
Treatment of hyperthyroidism	
No current therapy (n, %)	17 (32.1)
Antithyroid drugs (n, %)	28 (52.8)
Currently on oral antithyroid drug therapy (n, %)	23 (43.4)
MMI (mg/d)	0.00 (0.00, 1.56)
RAI therapy (n, %)	8 (15.1)
Hypothyroidism occurs after RAI therapy (n, %)	5 (62.5)
Thyroid surgery (n, %)	0 (0.0)
History of hypothyroidism (n, %)	16 (18.6)
Currently on levothyroxine treatment (n, %)	14 (87.5)
Levothyroxine (μg/d)	0 (0, 0)
History of thyroid malignancy (n, %)	7 (8.1)
Combined HT (n, %)	34 (39.5)
Combined hypertension (n, %)	11 (12.8)
Combined diabetes (n, %)	9 (10.5)
Smoker (n, %)	22 (25.6)
No immunosuppressive therapy in the last 3 months (n, %)	58 (67.4)

Values are presented as mean ± SD or median (25th percentile, 75th percentile), or number, percentage (n, %), according to distribution; Smoker was defined as having smoked more than 100 cigarettes in a lifetime. Abbreviations: TED denotes thyroid eye disease; MMI methimazole; RAI radioactive iodine.

**Table 2:** Clinical characteristics of 86 TED patients.

Parameter	TED patients (n = 86)
CAS	1.64 ± 1.28
Spontaneous orbital pain (n, %)	6 (7.0)
Gaze-evoked orbital pain (n, %)	5 (5.8)
Eyelid erythema (n, %)	9 (10.5)
Eyelid edema (n, %)	62 (72.1)
Conjunctival redness (n, %)	30 (37.2)
Chemosis (n, %)	11 (12.8)
Caruncle edema (n, %)	16 (18.6)
CAS ≥ 3 points (n, %)	20 (23.3)
Thyroid status	
Euthyroidism (n, %)	57 (66.3)
Hyperthyroidism (n, %)	18 (20.9)
Hypothyroidism (n, %)	10 (11.6)
FT3 (pmol/L)	5.18 ± 0.77
FT4 (pmol/L)	11.65 ± 2.24
TSH (mIU/L)	1.96 (1.03, 2.94)
TRAb (IU/ml)	1.17 ± 0.34
TGAb (IU/ml)	0.21 (0.01, 3.73)
TPOAb (IU/ml)	3.20 (0.64, 17.27)
TPOAb (+) (n, %)	28 (32.6)
TGAb (+) (n, %)	20 (23.3)
TPOAb (+) and TGAb (+) (n, %)	14 (16.3)
TPOAb (+) and TGAb (-) (n, %)	14 (16.3)
TPOAb (-) and TGAb (+) (n, %)	6 (6.9)
TPOAb (-) and TGAb (-) (n, %)	52 (60.5)
EUGOGO classification of severity of TED	
Mild (n, %)	20 (23.3)
Moderate-to-severe (n, %)	63 (73.3)
Sight-threatening (n, %)	3 (3.5)
DON (n, %)	3 (3.5)
Corneal ulcer (n, %)	0 (0.0)
NOSPECS classification	4.13 ± 1.24
Bilateral (n, %)	74 (86.0)
Eyelid retraction (n, %)	52 (60.5)
Proptosis (n, %)	72 (83.7)
Lagophthalmos (n, %)	20 (23.3)
Visual acuity	0.91 ± 0.24
Exophthalmos (mm)	19.22 ± 3.28
Gorman diplopia score	2 (0, 3)

Values are presented as mean ± SD or median (25th percentile, 75th percentile), or number, percentage (n, %), according to distribution; Abbreviations: TED denotes thyroid eye disease; CAS clinical activity score; FT3 Free Triiodothyronine; FT4 Free Thyroxine; TSH Thyroid Stimulating Hormone; TRAb thyrotrophin receptor antibody; TGAb thyroid globulin antibodies; TPOAb thyroid peroxidase antibodies; DON dysthyroid optic neuropathy.

## 3 Results

### 3.1 Demographic and clinical characteristics of 86 TRAb-negative TED patients

A total of 86 TRAb-negative TED patients met the inclusion criteria, with a mean age of 45.24 ± 10.78 years and a female-to-male ratio of 1.15:1. Twenty-two patients (25.6%) were smokers, with mean TED duration of 23.5 months (IQR 10.0–40.3) months. Fifty-eight patients (67.4%) did not receive immunosuppressive therapy within 3 months. Hyperthyroidism with a TRAb-negative history was present in 53 patients (61.6%), including 8 (15.1%) who received radioiodine (RAI) treatment (5 subsequently developed hypothyroidism). At admission, 57 patients (66.3%) were euthyroid. Seven patients (8.1%) had thyroid malignancy history with surgical treatment (**Tables 1**, **2**).

Bilateral ocular involvement was present in 74 patients (86.0%), with the more severely affected eye analyzed. Predominant manifestations included proptosis (83.7%), eyelid edema (72.1%), and eyelid retraction (60.5%). Mean CAS was 1.64 ± 1.28, with active disease in 20 patients (23.3%). EUGOGO classification identified moderate-to-severe disease in 63 patients (73.3%). Mean exophthalmos measured 19.22±3.28mm, visual acuity 0.91 ± 0.24, and Gorman diplopia score 2 (IQR 0–3). Antibody profiles showed mean TRAb 1.17 ± 0.34 IU/mL, TGAb 0.21 (IQR 0.02–3.73) IU/mL, and TPOAb 3.20 (IQR 0.64–17.27) IU/mL. TGAb positivity occurred in 20 patients (23.3%), TPOAb in 28 (32.6%), and both in 14 (16.3%) (**Table 2**).

### 3.2 Comparison of clinical characteristics of patients grouped based on TPOAb and TGAb

**Figure 2:**
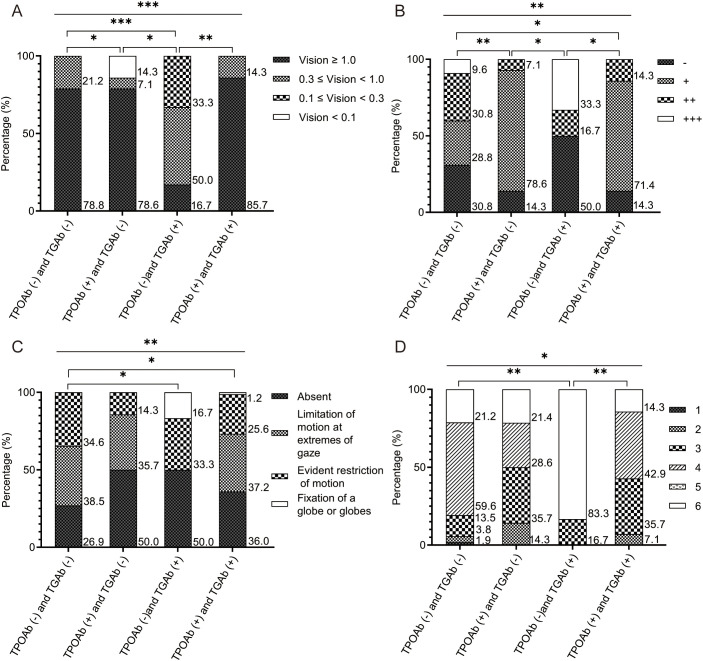
The comparison results of the four groups of patients with TED, based on TPOAb and TGAb. **(A)** Comparison results of visual acuity among the four groups. The number of patients with visual acuity ≥ 1 was the lowest in the TPOAb **(-)** and TGAb (+) group; **(B)** Comparison results of orbital pressure among the four groups. The proportion of patients with orbital pressure of ++ was the highest in the TPOAb **(-)** and TGAb (+) group; **(C)** Comparison results of soft tissue involvement among the four groups. The proportion of patients with fixed eyeballs or fixed eyeball clusters was the highest in the TPOAb (-) and TGAb (+) group; **(D)** Comparison results of NOSPECS classification among the four groups. The proportion of patients with NOSPECS classification of 6 or above was the highest in the TPOAb (-) and TGAb (+) group. The x-axis represents each group. The y-axis represents the percentage of the different groups’ population within each group. P values were showed as: *p < 0.05; **p < 0.01; ***p < 0.001. TED, thyroid eye disease. TPOAb, thyroid peroxidase antibody. TGAb, thyroglobulin antibody.

Eighty-six patients were stratified into four groups based on TPOAb/TGAb status: double-negative [TPOAb(-)/TGAb(-)], TPOAb(+)/TGAb(-), TPOAb(-)/TGAb(+), and double-positive [TPOAb(+)/TGAb(+)]. Baseline characteristics showed no significant differences (**Supplementary Table S1**). However, significant intergroup variations emerged in eyelid erythema and lagophthalmos (both p < 0.05). The TPOAb(-)/TGAb(+) and double-positive groups exhibited higher eyelid erythema incidence than other groups (p < 0.001). The TPOAb(-)/TGAb(+) group demonstrated the highest lagophthalmos frequency among all groups (p < 0.05), along with the lowest thyroglobulin levels (**Table 3**). This group also featured fewer patients with visual acuity of 1 or higher (**Figure 2A**), a higher proportion with +++ orbital pressure (**Figure 2B**), more cases of orbital fixation cases (**Figure 2C**), and a greater frequency of NOSPECS grade or higher (**Figure 2D**). Collectively, the TPOAb(-)/TGAb(+) group exhibited the most severe disease profile, characterized by worse NOSPECS grading, reduced visual acuity, orbital pressure, and soft tissue involvement. Analysis of isolated antibody effects revealed distinct clinical associations: TPOAb-positive patients showed increased proptosis (p < 0.05) but less active TED, eyelid retraction, and soft tissue involvement (p < 0.05). Intraocular pressure in this group was predominantly mildly elevated (+) (**Supplementary Table S2**). TGAb-positive patients presented higher incidences of eyelid erythema and visual impairment incidence but less diplopia (p < 0.05) (**Supplementary Table S3**).

**Table 3:** Clinical characteristics of 86 TED patients compared by TPOAb and TGAb.

Clinical characteristics	TPOAb (-) and TGAb (-)	TPOAb (+) and /or TGAb (+)			*P*-value
		TPOAb (+) and TGAb (-)	TPOAb (-) and TGAb (+)	TPOAb (+) and TGAb (+)	
N	52	14	6	14	
CAS ≥ 3 points (n, %)	16 (80.0)	0 (0.0)	2 (33.3)	2 (14.3)	0.077
CAS	1.73 ± 1.36	1.07 ± 0.73	2.00 ± 2.10	1.71 ± 0.91	0.321
Spontaneous orbital pain (n, %)	4 (7.7)	0 (0.0)	1 (16.7)	1 (7.1)	0.581
Gaze-evoked orbital pain (n, %)	5 (9.6)	0 (0.0)	0 (0.0)	0 (0.0)	0.325
Eyelid erythema (n, %)	2 (3.8)	0 (0.0)	2 (33.3) *^#^	5 (35.5) *^#^	0.001
Eyelid edema (n, %)	38 (73.1)	11 (78.6)	3 (50.0)	10 (71.4)	0.620
Conjunctival redness (n, %)	23 (44.2)	2 (14.3)	2 (33.3)	5 (35.7)	0.231
Chemosis (n, %)	7 (13.5)	1 (7.1)	2 (33.3)	1 (7.1)	0.378
Caruncle edema (n, %)	11 (21.2)	1 (7.1)	2 (33.3)	2 (14.3)	0.481
Bilateral (n, %)	45 (86.5)	12 (85.7)	4 (66.7)	13 (92.9)	0.488
Eyelid retraction (n, %)	34 (65.4)	6 (42.9)	5 (83.3)	7 (50.0)	0.231
Proptosis (n, %)	41 (78.8)	13 (92.9)	4 (66.7)	14 (100)	0.124
EUGOGO classification of severity of TED					
Mild (n, %)	14 (26.9)	3 (21.4)	1 (16.7)	2 (14.3)	0.462
Moderate-to-severe (n, %)	37 (71.2)	10 (71.4)	4 (66.7)	12 (85.7)	
Sight-threatening (n, %)	1 (1.9)	1 (7.1)	1 (16.7)	0 (0.0)	
DON (n, %)	1 (1.9)	1 (7.1)	1 (16.7)	0 (0.0)	0.209
Lagophthalmos (n, %)	12 (23.1)	4 (28.6)	4 (66.7)	0 (0.0) *^†^	0.013
Exophthalmos (mm)	18.94 ± 3.60	19.14 ± 1.96	19.50 ± 4.93	20.21 ± 2.19	0.641
Gorman diplopia score	2.0 (0.0, 3.0)	1.0 (0.0, 3.0)	0.5 (0.0, 1.5)	1.0 (0.0, 2.0)	0.205
Thyroid status					0.073
Euthyroidism (n, %)	36 (69.2)	10 (71.4)	4 (66.7)	8 (57.1)	
Hyperthyroidism (n, %)	12 (23.1)	4 (28.6)	1 (16.7)	1 (7.1)	
Hypothyroidism (n, %)	4 (7.7)	0 (0.0)	1 (16.7)	5 (35.7)	
FT3 (pmol/L)	5.13 ± 0.61	5.25 ± 1.23	5.33 ± 0.85	5.14 ± 0.73	0.766
FT4 (pmol/L)	11.64 ± 2.24	11.45 ± 1.98	12.32 ± 4.05	11.63 ± 1.59	0.889
TSH (mIU/L)	1.54 (0.68, 2.79)	2.59 (2.04, 3.29)	2.26 (1.31, 10.04)	2.50 (1.49, 6.43)	0.059
TRAb (IU/ml)	1.19 ± 0.35	0.96 ± 0.20	1.23 ± 0.36	1.29 ± 0.37	0.052
TG (ng/ml)	11.02 (4.51, 20.02)	7.31 (0.74, 17.68)	0.10 (0.05, 0.95) *^#^	0.72 (0.25, 4.05) *	< 0.001

*, #, and † indicated P < 0.05 for comparisons TPOAb (-) and TGAb (-) group, TPOAb (+) and TGAb (-) group, and TPOAb (-) and TGAb (+) group. ※ represent that the contrast differences among the 4 groups of TED patients are statistically significant. Abbreviations: TED denotes thyroid eye disease; FT3 Free Triiodothyronine; FT4 Free Thyroxine; TRAb thyrotrophin receptor antibody; TGAb thyroid globulin antibodies; TPOAb thyroid peroxidase antibodies; CAS clinical activity score; TSH Thyroid Stimulating Hormone; DON dysthyroid optic neuropathy; TG thyroglobulin.

### 3.3 Thyroid-related antibodies in TRAb-negative TED patients were significantly correlated with clinical characteristics

**Figure 3:**
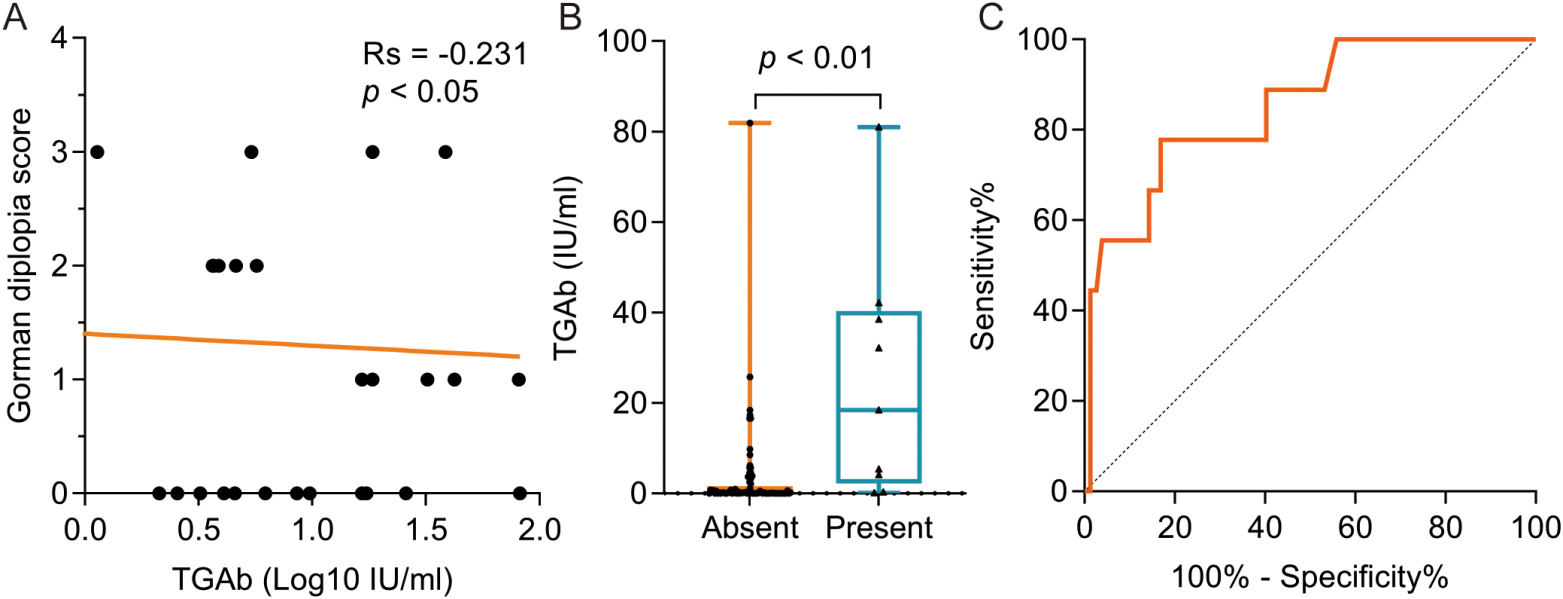
The correlation between TGAb and clinical characteristics in TRAb-negative TED Patient. **(A)** The negative correlation was evident between TGAb and Gorman diplopia score; **(B)** The TGAb level was significantly higher in the group with eyelid erythema symptoms compared to the group without symptoms at 18.39 (2.18, 40.32) IU/ml and 0.18 (0.00, 1.63) IU/ml, respectively, p < 0.01; **(C)** ROC curve analysis was performed using TGAb as the dependent variable and the presence of eyelid erythema as the outcome variable, yielding an AUC of 0.851 (95% CI: 0.721 – 0.980), p < 0.001, sensitivity of 0.778, specificity of 0.831, and a cutoff value of 3.980 IU/ml. TED, thyroid eye disease. TPOAb, thyroid peroxidase antibody. TGAb, thyroglobulin antibody. ROC, receiver operating characteristic. AUC, area under the curve.

Spearman correlation analysis revealed a significant negative association between TGAb levels and Gorman diplopia scores (Rs = -0.231, p < 0.05; **Figure 3A**). Patients with eyelid erythema demonstrated significantly higher TGAb levels compared to those without this manifestation (p < 0.01; **Figure 3B**). Multivariate logistic regression adjusted for age, sex, history of hyperthyroidism history, antithyroid therapy (medication/RAI), TED duration, smoking status, and recent immunosuppressive therapy identified TGAb as an independent risk factor for eyelid erythema (**Table 4**). ROC analysis using TGAb to predict eyelid erythema yielded an AUC of 0.851 (95% CI: 0.721-0.980; p < 0.001). The optimal cutoff of 3.980 IU/mL yielded a sensitivity of 0.778 and a specificity of 0.831 (**Figure 3C**).

**Table 4:** TGAb was a risk factor for eyelid erythema.

Risk factor	Odds ratio (OR)	95% confidence interval (95% CI)	*P-*value
Model 1			
TGAb	1.064	1.017 – 1.114	0.008*
Model 2			
TGAb	1.116	1.031 – 1.208	0.007*
Age (years)	1.138	0.997 – 1.300	0.055
Gender (f/m)	0.854	0.063 – 11.592	0.906
History of hyperthyroidism	58.377	1.734 – 1964.804	0.023*
Antithyroid therapy (including drug and RAI therapy)	0.074	0.005 – 1.014	0.051
TED duration (months)	1.000	0.979 – 1.022	0.978
Smoker	0.793	0.037 – 17.190	0.882
Immunosuppressive therapy in the last 3 months	2.755	0.229 – 33.075	0.424

Model 1 is a univariate logistic regression analysis with eyelid erythema as the dependent variable. Model 2 is a multivariate logistic regression analysis adjusted for age, gender, history of hyperthyroidism, antithyroid therapy (including drug and RAI therapy), TED duration, smoker, and immunosuppressive therapy in the last 3 months with eyelid erythema as the dependent variable. *P < 0.05.

Notably, TRAb negativity does not imply absolute absence of TRAb. Within detectable ranges (0.8 - 1.75 IU/ml), TRAb levels showed a positive correlation with CAS (Rs = 0.377, p < 0.01). Patients exhibiting conjunctival injection and caruncle edema had higher TRAb levels than asymptomatic counterparts (p < 0.01; **Supplementary Figures S1A, B**). TRAb independently predicted these manifestations (p < 0.05; **Supplementary Table S4**) with significant ROC analysis (**Supplementary Figure S1C**).

Collectively, TRAb-negative TED presents distinct clinical features. Both TPOAb and TGAb associated with key ocular manifestations including proptosis, lagophthalmos, and elevated orbital pressure. Notably, TGAb serves as an effective predictor for eyelid erythema, highlighting the clinical necessity of monitoring these antibodies in TRAb-negative TED evaluation and management.

## 4 Discussion

This cross-sectional observational study comprehensively characterized 86 TRAb-negative TED patients, establishing predictive thresholds for specific symptoms such as eyelid erythema, conjunctival injection, and caruncle edema.

Our cohort accounted for 12.4% (86/692) of TED cases, consistent with the reported prevalence (12.0-19.4%) (14, 15). The mean age was 45.24 ± 10.78 years with 53.5% of participants being female (female-to-male ratio 1.15), aligning with demographic patterns (11, 16). Bilateral involvement occurred in 86.0% (74/86) of patients, with the most common clinical signs being proptosis (83.7%), eyelid edema (72.1%), and eyelid retraction (60.5%). While proptosis and eyelid retraction represent common features of TED features (11, 17), our cohort demonstrated higher prevalence of eyelid edema prevalence than HT-associated TED (18). Moderate-to-severe disease affected 73.3% of patients, exceeding rates in HT-TED cohorts (19) but consistent with other TRAb-negative populations (6), potentially reflecting racial/geographic variations (20, 21). Vision-threatening dysthyroid optic neuropathy (DON) occurred in 3.5%, comparable to prior reports (3.2%) (16).

Although thyroid-stimulating immunoglobulins (TSI) demonstrate superior diagnostic performance (22), their clinical implementation remains limited. Therefore, the third-generation electrochemiluminescent-based TRAb assay used in this study, represents the most widely adopted clinical standard (23). While TSH receptor (TSHR)-mediated orbital inflammation remains central to TED pathogenesis (24, 25), our findings suggest that alternative mechanisms may be operational in TRAb-negative patients. The TPOAb(-)/TGAb(+) subgroup exhibited severe manifestations including eyelid erythema, lagophthalmos, visual impairment, elevated orbital pressure, and advanced NOSPECS grading. Contrasting reports of TGAb’s protective role in GD-TED (26), our non-GD cohort demonstrated TGAb positivity associated with multiple severe symptoms. This apparent discrepancy likely stems from fundamental differences in the underlying autoimmune pathogenesis between GD and non-GD (TRAb-negative) populations. In TRAb-negative TED, TGAb positivity may reflect a distinct pathogenic process involving synergistic action with other antibodies, direct effects on orbital tissue effects, or serve as an indicator of heightened inflammation. The particularly severe manifestations in the TPOAb(-)/TGAb(+) subgroup further suggest that the absence of potentially immunomodulatory TPOAb (27, 28) may unmask a more severe disease associated with TGAb in this setting. However, these mechanisms remain speculative, and our study design cannot establish a causal relationship. Unmeasured confounding factors related to the distinct etiologies within TRAb-negative TED could also contribute to the observed association. Future mechanistic studies using cellular models to assess TGAb’s effects on orbital fibroblasts, exploration in relevant animal models, and longitudinal assessments in well-characterized TRAb-negative TED cohorts are crucial to validate these findings and elucidate the precise role of TGAb in this specific TED phenotype. After adjusting for confounders (age, sex, history of hyperthyroidism history, antithyroid therapy [medication/RAI], TED duration, smoking status, and recent immunosuppression) (2, 15), multivariate analysis confirmed TGAb as an independent risk factor for eyelid erythema (optimal cutoff: 3.980 IU/mL). TPOAb positivity correlated with reduced active TED, eyelid retraction, and soft tissue involvement - potentially reflecting orbital TPO-mediated immunomodulation (27, 28).

Pathogenesis extends beyond TSHR autoimmunity, involving potential roles for: eye muscle antigens (calsequestrin, collagen XIII) (29–31); Vitamin D deficiency (32); Müller’s muscle autoimmunity (33); and shared thyroid-orbital epitopes (G2s protein) (34). Novel findings include a negative correlation between the TGAb-Gorman diplopia score and Rs (-0.231, p < 0.05), necessitating a mechanistic investigation. Detectable subthreshold TRAb levels positively correlated with CAS (Rs=0.377, p<0.01) and predicted conjunctival injection/caruncle edema, suggesting that clinical management may require achieving lower TRAb thresholds beyond mere negativity. The limitations of this study include: a mean disease duration of >18 months (32.6% received recent immunosuppression); potential recall bias regarding historical TRAb status; and a modest sample size despite representing the largest TRAb-negative TED cohort reported. Additionally, while TGAb and TPOAb levels correlated significantly with specific clinical features, they do not imply causation, as unmeasured confounders may influence these associations. Future longitudinal studies and mechanistic investigations using cellular/animal models are warranted to validate these findings and elucidate pathogenesis.

## 5 Conclusions

The main clinical manifestations in TRAb-negative TED patients included proptosis, eyelid edema, and eyelid retraction. TGAb and TPOAb levels were significantly associated with specific clinical features, necessitating routine monitoring of these antibodies in TRAb-negative TED management. The TSHR-mediated pathogenesis cannot fully explain all ocular manifestations, warranting investigation of additional pathogenic mechanisms. Systematic assessment of ocular characteristics—particularly in TRAb-negative patients with Hashimoto’s thyroiditis—enables the earlier recognition of TED recognition and the implementation of preventive interventions.

## Data Availability

The original contributions presented in the study are included in the article/**Supplementary Material**. Further inquiries can be directed to the corresponding authors.

## Supplementary material

Supplementary Figure 1 The correlation between TRAb and Clinical Characteristics in TRAb-Negative TED Patients. **(A)** The TRAb level was significantly higher in the group with conjunctival redness symptoms compared to the group without symptoms at 1.33 ± 0.36 IU/ml and 1.08 ± 0.29 IU/ml, IU/ml, respectively, p < 0.01; **(B)** The TRAb level was significantly higher in the group with caruncular oedema symptoms compared to the group without symptoms, at 1.39 ± 0.28 IU/ml and 1.12 ± 0.34 IU/ml, respectively, p < 0. 01; **(C)** ROC curve analysis was performed using TRAb as the dependent variable and conjunctival redness and caruncle edema, yielding AUCs of 0.708 (95% CI: 0.589 – 0.827, p < 0. 01) for conjunctival congestion, sensitivity of 0.406, specificity of 0.944 and a cutoff value of 1.580 IU/ml; and 0.711 (95% CI: 0.587 – 0.836, p < 0.01) for lacrimal duct swelling, sensitivity of 0.875, specificity of 0.586 and a cutoff value of 1.155 IU/ml.
