# Supplementary figures and images for: Clinical profiling of TPOAb and TGAb in patients with thyrotrophin receptor antibody-negative thyroid eye disease: A single-center observational study in China
Source: Front Endocrinol (Lausanne). 2025 Sep 22;16:1655598. doi: 10.3389/fendo.2025.1655598 (PMC12497607; doi:10.3389/fendo.2025.1655598)

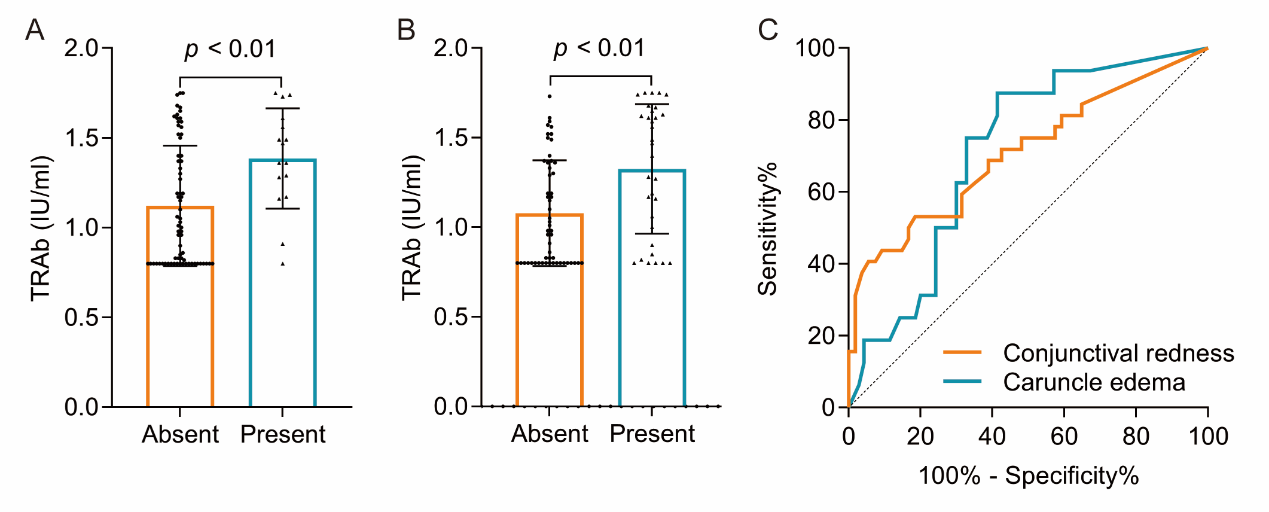

Supplement: Supplementary Figure 1 — The correlation between TRAb and Clinical Characteristics in TRAb-Negative TED Patients. (A) The TRAb level was significantly higher in the group with conjunctival redness symptoms compared to the group without symptoms at 1.33 ± 0.36 IU/ml and 1.08 ± 0.29 IU/ml, IU/ml, respectively, p < 0.01; (B) The TRAb level was significantly higher in the group with caruncular oedema symptoms compared to the group without symptoms, at 1.39 ± 0.28 IU/ml and 1.12 ± 0.34 IU/ml, respectively, p < 0. 01; (C) ROC curve analysis was performed using TRAb as the dependent variable and conjunctival redness and caruncle edema, yielding AUCs of 0.708 (95% CI: 0.589 – 0.827, p < 0. 01) for conjunctival congestion, sensitivity of 0.406, specificity of 0.944 and a cutoff value of 1.580 IU/ml; and 0.711 (95% CI: 0.587 – 0.836, p < 0.01) for lacrimal duct swelling, sensitivity of 0.875, specificity of 0.586 and a cutoff value of 1.155 IU/ml. [file Image1.tif]
