# Supplementary material for: Clinical profiling of TPOAb and TGAb in patients with thyrotrophin receptor antibody-negative thyroid eye disease: A single-center observational study in China
Source: Front Endocrinol (Lausanne). 2025 Sep 22;16:1655598. doi: 10.3389/fendo.2025.1655598 (PMC12497607; doi:10.3389/fendo.2025.1655598)
Supplement: Supplementary file 2 [file Table1.docx]

**Supplementary Table S1. Four groups of the 86 TED patients with highly consistent baseline features**

| Clinical characteristics | TPOAb (-) and TGAb (-) | TPOAb (+) and /or TGAb (+) | | | *P*-value |
| --- | --- | --- | --- | --- | --- |
|  |  | TPOAb (+) and TGAb (-) | TPOAb (-) and TGAb (+) | TPOAb (+) and TGAb (+) |  |
| N | 52 | 14 | 6 | 14 |  |
| Age (years) | 46.54 ± 9.79 | 41.86 ± 9.87 | 49.83 ± 14.62 | 41.86±12.79 | 0.204 |
| Gender (m/f, n, %) | 24 (46.2)/28 (53.8) | 6 (42.9)/8 (57.1) | 2 (33.3)/4 (66.7) | 8(57.1)/6(42.9) | 0.769 |
| History of hyperthyroidism (n, %) | 30 (57.7) | 10 (71.4) | 2 (33.3) | 11(78.6) | 0.200 |
| Hyperthyroidism duration (months) | 6.50 (0.00, 35.50) | 21.50 (0.00,97.50) | 0.00 (0.00, 18.00) | 29.00 (1.50, 131.25) | 0.103 |
| TED duration (months) | 24.00 (13.25, 46.75) | 28.50 (8.25, 48.75) | 7.50 (6.00, 24.00) | 15.00(11.25, 42.25) | 0.238 |
| History of hypothyroidism (n, %) | 13 (25.0) | 2 (14.3) | 1 (16.7) | 0 (0.0) | 0.188 |
| Received immunosuppressive therapy in the last 3 months (n, %) | 18 (34.6) | 5 (35.7) | 3 (50.0) | 2 (14.3) | 0.373 |
| History of thyroid malignancy (n, %) | 7 (13.5) | 0 (0.0) | 0 (0.0) | 0 (0.0) | 0.184 |
| History of RAI therapy (n, %) | 4 (7.7) | 2 (14.3) | 1 (16.7) | 1 (7.1) | 0.793 |
| Smoker (n, %) | 12 (23.1) | 3 (21.4) | 2 (33.3) | 5 (35.7) | 0.743 |

Notes. According to distribution, values are presented as mean ± SD or median (25th percentile, 75th percentile), or number, percentage (n, %); Smoker was defined as having smoked more than 100 cigarettes in a lifetime. Abbreviations: TED denotes thyroid eye disease, GD Graves’ disease, RAI radioactive iodine.
