# Supplementary material for: Clinical profiling of TPOAb and TGAb in patients with thyrotrophin receptor antibody-negative thyroid eye disease: A single-center observational study in China
Source: Front Endocrinol (Lausanne). 2025 Sep 22;16:1655598. doi: 10.3389/fendo.2025.1655598 (PMC12497607; doi:10.3389/fendo.2025.1655598)
Supplement: Supplementary file 3 [file Table2.docx]

**Supplementary Table S2****. Comparison of clinical characteristics between TPOAb-positive and TPOAb-negative individuals**

| Clinical characteristics | TPOAb-negative  (n=58) | TPOAb-positive  (n=28) | *P*-value |
| --- | --- | --- | --- |
| Activity |  |  | 0.046* |
| Active (n, %) | 41 (70.7) | 25 (89.3) |  |
| Inactive (n, %) | 17 (29.3) | 3 (10.7) |  |
| Eyelid retraction (n, %) |  |  | 0.019* |
| Absent (n, %) | 18 (31.0) | 16 (57.1) |  |
| Present (n, %) | 40 (69.0) | 12 (42.9) |  |
| Soft tissue involvement |  |  | 0.035* |
| Absent (n, %) | 17 (29.3) | 14 (50.0) |  |
| Limitation of motion at extremes of gaze | 20 (34.5) | 12 (42.9) |  |
| Evident restriction of motion | 20 (34.5) | 2 (7.1) |  |
| Fixation of a globe or globes | 1 (1.7) | 0 (0.0) |  |
| Proptosis |  |  | 0.022* |
| Absent (n, %) | 13 (22.4) | 1 (3.6) |  |
| Present (n, %) | 45 (77.6) | 27 (96.4) |  |
| Orbital pressure |  |  | 0.000* |
| - (n, %) | 19 (32.8) | 4 (14.3) |  |
| + (n, %) | 15 (25.9) | 21 (75.0) |  |
| ++ (n, %) | 17 (29.3) | 3 (10.7) |  |
| +++ (n, %) | 7 (12.1) | 0 (0.0) |  |

Notes. **P* < 0.05; Abbreviations: TPOAb denotes thyroid peroxidase antibodies.
