# Supplementary material for: Clinical profiling of TPOAb and TGAb in patients with thyrotrophin receptor antibody-negative thyroid eye disease: A single-center observational study in China
Source: Front Endocrinol (Lausanne). 2025 Sep 22;16:1655598. doi: 10.3389/fendo.2025.1655598 (PMC12497607; doi:10.3389/fendo.2025.1655598)
Supplement: Supplementary file 4 [file Table3.docx]

**Supplementary Table S3. Comparison of clinical characteristics between TGAb-positive and TGAb-negative individuals**

| Clinical characteristics | TGAb-negative  (n=66) | TGAb-positive  (n=20) | *P*-value |
| --- | --- | --- | --- |
| Eyelid erythema |  |  | 0.000* |
| Absent (n, %) | 64 (97.0) | 13 (65.0) |  |
| Present (n, %) | 2 (3.0) | 7 (35.0) |  |
| Visual acuity |  |  | 0.047* |
| Vision ≥ 1 | 52 (78.8) | 13 (65.0) |  |
| 0.3 ≤ Vision < 1 | 12 (18.2) | 5 (25.0) |  |
| 0.1 ≤ Vision < 0.3 | 0 (0.0) | 2 (10.0) |  |
| Vision < 0.1 | 2 (3.0) | 0 (0.0) |  |
| Gorman diplopia score |  |  | 0.001* |
| 0 (n, %) | 24 (36.4) | 9 (45.0) |  |
| 1 (n, %) | 1 (1.5) | 5 (25.0) |  |
| 2 (n, %) | 14 (21.2) | 3 (15.0) |  |
| 3 (n, %) | 27 (40.9) | 3 (15.0) |  |

Notes. **P* < 0.05; Abbreviations: TGAb denotes thyroid globulin antibodies.
