# Supplementary material for: Clinical profiling of TPOAb and TGAb in patients with thyrotrophin receptor antibody-negative thyroid eye disease: A single-center observational study in China
Source: Front Endocrinol (Lausanne). 2025 Sep 22;16:1655598. doi: 10.3389/fendo.2025.1655598 (PMC12497607; doi:10.3389/fendo.2025.1655598)
Supplement: Supplementary file 5 [file Table4.docx]

**Supplementary Table S4. TRAb was a risk factor for conjunctival redness and caruncular oedema**

| Risk factor | Odds ratio  (OR) | 95% confidence interval (95% CI) | *P-*value |
| --- | --- | --- | --- |
| Model 1 |  |  |  |
| TRAb | 9.491 | 2.324 – 38.753 | 0.002* |
| Model 2 |  |  |  |
| TRAb | 6.889 | 1.405 – 33.779 | 0.017* |
| Age (years) | 1.013 | 0.961 – 1.069 | 0.628 |
| Gender (f/m) | 0.290 | 0.080 – 1.046 | 0.059 |
| History of hyperthyroidism | 5.985 | 1.283 – 27.916 | 0.023* |
| Antithyroid therapy (including drug and RAI therapy) | 0.704 | 0.185 – 2.677 | 0.704 |
| TED duration (months) | 1.000 | 0.991 – 1.009 | 0.964 |
| Smoker | 0.513 | 0.108 – 2.428 | 0.400 |
| Immunosuppressive therapy in the last 3 months | 0.661 | 0.200 – 2.186 | 0.497 |
| Model 3 |  |  |  |
| TRAb | 10.888 | 1.871 – 63.353 | 0.008* |
| Model 4 |  |  |  |
| TRAb | 7.667 | 1.121 – 52.429 | 0.038* |
| Age (years) | 1.017 | 0.950 – 1.090 | 0.622 |
| Gender (f/m) | 0.894 | 0.222 – 3.713 | 0.894 |
| History of hyperthyroidism | 1.090 | 0.169 – 7.042 | 0.928 |
| Antithyroid therapy (including drug and RAI therapy) | 1.369 | 0.260 – 7.203 | 0.711 |
| TED duration (months) | 1.003 | 0.993 – 1.013 | 0.572 |
| Smoker | 1.071 | 0.160 – 7.164 | 0.943 |
| Immunosuppressive therapy in the last 3 months | 0.115 | 0.013 – 1.053 | 0.056 |

Notes. Model 1 and Model 3 are univariate logistic regression analysis with conjunctival redness and caruncular oedema as the dependent variables, respectively. Model 2 and Model 4 are multivariate logistic regression analysis adjusted for age, gender, history of hyperthyroidism, antithyroid therapy (including drug and RAI therapy), TED duration, smoker, and immunosuppressive therapy in the last 3 months with conjunctival redness and caruncular oedema as the dependent variables, respectively; **P* < 0.05.
